# A high throughput gold nanoparticles chemiluminescence detection of opioid receptor antagonist naloxone hydrochloride

**DOI:** 10.1186/s13065-015-0083-6

**Published:** 2015-02-11

**Authors:** Nawal A Alarfaj, Maha F El-Tohamy

**Affiliations:** Department of Chemistry, College of Science, King Saud University, P.O. Box 22452, Riyadh, 11495 Saudi Arabia; Permanent address: General Administrative of Medical Affairs, Zagazig University, Zagazig, Egypt

**Keywords:** Chemiluminescence, Gold nanoparticles, Naloxone hydrochloride, Luminol, Potassium ferricyanide, Pharmaceutical dosage forms, Biological fluids

## Abstract

**Background:**

The opioid antagonist agent naloxone hydrochloride (NLX) is a drug that has high affinity for opiate receptors but do not activate these receptors. Owing to the role of this drug to block the effects of exogenous administered opioids and endogenous released endorphians we can deduce the importance of developing sensitive analytical methods for detection of such drug. In the present study gold nanoparticles (AuNPs) was employed for enhancing the chemiluminescence (CL) signals arising from luminol-ferricyanide reaction in the presence of naloxone hydrochloride using sequential injection chemiluminescence analysis (SIA).

**Method:**

In the present study gold nanoparticles (AuNPs) was employed for enhancing the chemiluminescence (CL) signals arising from luminol-ferricyanide reaction in the presence of naloxone hydrochloride using sequential injection chemiluminescence analysis (SIA).

**Results:**

The developed method was examined under optimum experimental conditions and the obtained results revealed a linear relationship between the relative CL intensity and the investigated drug at a concentration range of 1.0×10^−9^-1.0×10^−2^ mol L^−1^, (r = 0.9993, n=9) with detection and quantification limits of 1.6×10^−11^ and 1.0×10^−9^ mol L^−1^, respectively. The relative standard deviation was 0.9%.

**Conclusion:**

The proposed method was employed for the determination of the investigated drug in bulk powder, its pharmaceutical dosage forms and biological fluids. The interference of some metals and amino acids on the CL intensity was investigated. Also the interference of some related pharmacological action drugs was tested. The obtained results of the developed method were statistically treated and compared with those obtained from other reported methods.

**Graphical Abstract:**
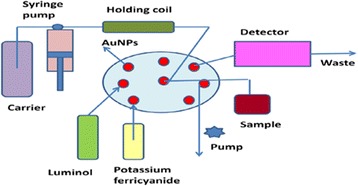
Utility of gold nanparticles in luminol-potassium ferricyanide chemiluminescence system for determination of naloxone hydrochloride.

## Background

Opioid antagonist such as a naloxone hydrochloride (NLX) is considered as an essential tool in scientific evaluations of opiates and opiate receptors [1]. It is clinically used after opioid overdoses or opioid anesthesia to restore spontaneous ventilation in patients who breathe inadequately. Also, it can reduce opioid- induced nausea and vomiting, pruritus, urinary retention, rigidity etc. [2].

**Figure 1:**
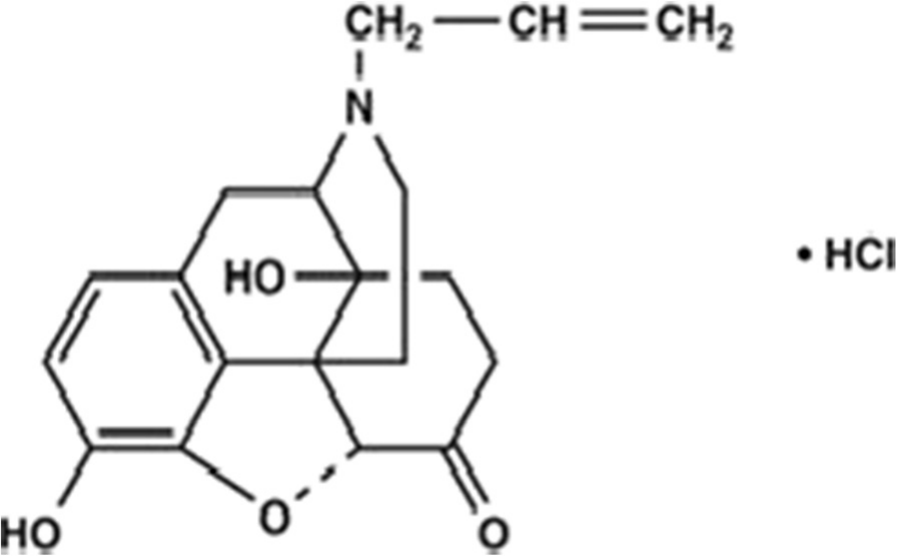
**Chemical structure of naloxone hydrochloride.**

Naloxone hydrochloride (Figure 1) is a drug that belongs to a group of medications known as narcotic antagonists. They are used to treat overdoses of narcotic medications (such as morphine, codeine, and oxycodone). It works by reversing the side effects of the narcotics, such as sedation and decreased breathing rate [2]. The literature survey reveals that several methods have been reported for determination of NLX mainly including high performance liquid chromatography [3-7], high performance liquid chromatography coupled with mass spectrometry [8-11], gas–liquid chromatography [12], chemiluminescence [13-15].

The chemiluminescence technique was based on the chemical reactions that emit light in the visible and infrared region without any need for excitation source [16]. The chemists were categorized the chemiluminescence to three types. Reactions involve highly oxidizied peroxide species, biochemiluminescence reactions which occure in living organisms and electrogenerated chemiluminescence reactions that used the electrical current to intiate the emitting light. Owing to the optical properties of the nanoparticles such as silver or gold nanoparticles, the researchers have been attended to use nanotechnology in many analytical methods, in particular the use of silver or gold nanoparticles to enhance various chemical chemiluminescence reactions [17,18]. They were found that the use of silver or gold nanoparticles in nanoscale size increased the intensity and the duration of the produced light. Additionally, due to the chemical stability and the high resistance of surface oxidation of gold nanoparticles rather than silver nanoparticles, the employing of gold nanoparticles in enhancing the chemiluminescence reactions were considered the point of view for many researchers [19].

The objective of the present study is to develop new, simple and highly sensitive sequential injection chemiluminescence analysis (SIA-CL) method using gold nanoparticles-luminol-ferricyanide system to determine naloxone hydrochloride an opioid antagonist receptor drug. The proposed method is more simple, less time and reagents consuming, highly sensitive with lower limit of detection than other chromatographic methods. The developed method conditionally optimized and employed for the detection of the investigated drug in bulk powder, its pharmaceutical dosage forms and biological fluids.

## Results and discussion

To select the optimum conditions for CL analysis various preliminary studies should be carried out. These include the selection of the oxidizing agent, the effect of alkaline medium, effect of luminol and potassium ferricyanide concentrations, effect of AuNPs concentration on the CL intensity and optimization of aspirated volume of reagents and samples, etc.

### Selection of potassium ferricyanide as oxidizing agent

**Figure 2:**
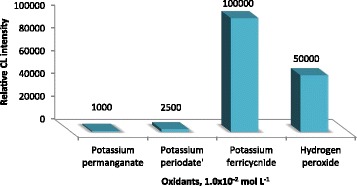
**Histogram of different kinds of oxidants on CL intensity of AuNPs-luminol system.**

Various oxidants including potassium ferricyanide, potassium permanganate, potassium periodate and hydrogen peroxide were investigated to select the most suitable oxidizing agents. No CL signals were detected by using potassium permanganate or potassium periodate. While, using hydrogen peroxide or potassium ferricyanide exhibit CL signals. As shown in Figure 2 potassium ferricyanide gave the higher CL intensity signal rather than that of hydrogen peroxide. Therefore, luminol-potassium ferricyanide-CL system was selected and the effect of luminol and potassium ferricyanide concentration was further investigated and optimized.

### Effect of luminol and potassium ferricyanide concentration

**Figure 3:**
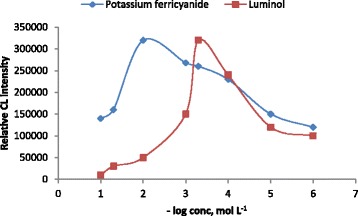
**Effect of luminol and potassium ferricyanide concentration on CL intensity, for luminol concentration (Au NPs 1.0×** **10** ^**−5**^ **mol L** ^**−1**^ **and potassium ferricyanide 1.0×** **10** ^**−2**^ **mol L** ^**−1**^ **) and for potassium ferricyanide concentration (Au NPs 1.0×** **10** ^**−5**^ **mol L** ^**−1**^ **, luminol 5.0×** **10** ^**−4**^ **mol L** ^**−1**^ **) and 1.0×** **10** ^**−3**^ **mol L** ^**−1**^ **naloxone hydrochloride.**

The effect of luminol and potassium ferricyanide concentrations on the CL signals was considered as one of the most important factors which should be investigated. Various concentration ranges of 1.0×10^−6^-1.0×10^−1^ mol L^−1^ of each luminol and potassium ferricyanide were tested. The CL signals were recorded and it was found that the CL signals were sharply increased in the presence of 5.0×10^−4^ mol L^−1^ of luminol and 1.0×10^−2^ mol L^−1^ of potassium ferricyanide. Therefore, the subsequent experimental analysis was carried out using these concentrations as indicated in (Figure 3).

### Effect of AuNPs concentration

**Figure 4:**
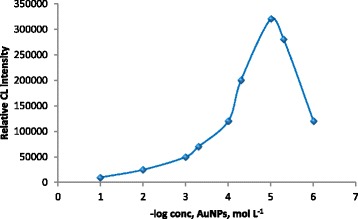
**Effect of AuNPs concentration on the CL signal of luminol-potassium ferricyanide system (luminol 5.0×** **10** ^**−4**^ **mol L** ^**−1**^ **, potassium ferricyanide 1.0×** **10** ^**−2**^ **mol L** ^**−1**^ **) and 1.0×** **10** ^**−3**^ **mol L** ^**−1**^ **naloxone hydrochloride.**

The CL intensity of luminol-potassium ferricyanide can be greatly affected by the concentration of AuNPs added to the system. The influence of AuNPs concentration was investigated over the concentration range 1.0×10^−6^-1.0×10^−1^ mol L^−1^. The suitable concentration of AuNPs was found to be 1.0×10^−5^ mol L^−1^ as shown in (Figure 4) and hence was selected in further studies.

### Optimization of alkaline medium

**Figure 5:**
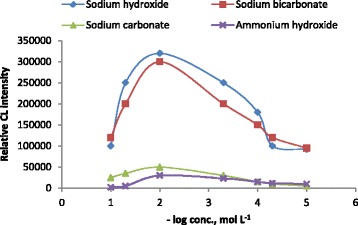
**Effect of sodium hydroxide, ammonium hydroxide, sodium carbonate and sodium bicarbonate concentration on CL intensity of luminol-potassium ferricyanide system (AuNPs 1.0×** **10** ^**−5**^ **mol L** ^**−3**^ **, potassium ferricyanide 1.0×** **10** ^**−2**^ **mol L** ^**−1**^ **, luminol 5.0×** **10** ^**−4**^ **mol L** ^**−1**^ **) and 1.0×** **10** ^**−3**^ **mol L** ^**−1**^ **naloxone hydrochloride.**

Owing to the reaction of luminol-ferricyanide in CL system should be optimized in of alkaline medium, four kinds of alkaline media, including ammonium hydroxide, sodium carbonate, sodium bicarbonate and sodium hydroxide in the range 1.0×10^−5^-1.0×10^−1^ mol L^−1^ were investigated. It was found that a sharp CL signal was obtained by using 1.0×10^−2^ mol L^−1^ sodium hydroxide. While the CL signals were significantly decreased by using the other three alkaline media, therefore 1.0×10^−2^ mol L^−1^ sodium hydroxide was used for preparation of luminol in the developed CL method (Figure 5).

### Optimization of aspirated volumes of sample and reagents

**Figure 6:**
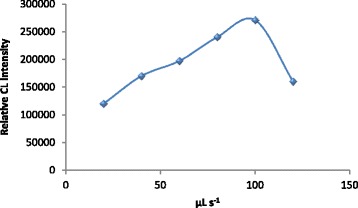
**The influence of flow rate on the relative CL intensity. Conditions; 50 μL of 5.0×** **10** ^**−4**^ **mol L** ^**−1**^ **luminol; 50 μL of 1.0×** **10** ^**−5**^ **AuNPs mol L** ^**−1**^ **, 30 μL of 1.0×** **10** ^**−2**^ **mol L** ^**−1**^ **ferricyanide and 30 μL of 1.0×** **10** ^**−3**^ **mol L** ^**−1**^ **naloxone hydrochloride.**

For AIA detection the aspirated volume of sample and reagents should be carefully optimized. FIAlab software version 5.9.321 was used for computer control and automatic optimization of the aspirated volume of both samples and reagents. The optimum aspirated volume for luminol, AuNPs and potassium ferricyanide was 50, 50 and 30 μL, respectively and 30 μL for NLX sample. The time for complete cycle was extended to about 45 s. The flow rate was tested in the range of 20–120 μL s^−1^. The optimum flow rate was found to be 100 μL s^−1^ and was used for further studies (Figure 6).

### SIA control program

The SIA control program was employed to perform all calibration measurements and experimental analysis of NLX. Also, the investigated drug was determined by utilizing an SIA control program in standard form, its pharmaceutical dosage forms and in biological fluids. The typical program was presented in Table 1. Each cycle was performed in 45 s and the throughput of samples can be recorded as 80 h^−1^.

**Table 1:** **The control program of AuNPs- luminol- potassium ferricyanide SIA-chemiluminescence detection of NLX**

**Device**	**Command**	**Parameter**	**Action**
**‘Loop Start (♯) 1**			
**Next sample**	**Counter clockwise**		
**Peristaltic pump**	**Delay (s)**	**% 50**	
	**Peristaltic pump off**	**25**	
**Detector**	**ON**		
**Syringe pump**	**Valve position IN**		
**Syringe pump**	**Set flow rate (μL s** ^**−1**^ **)**	**100**	
**Syringe pump**	**Aspirate (μL)**	**1500**	**Pump filled with carrier**
**Syringe pump**	**Delay until done**		
**Multiposition valve**	**Set valve position**	**3**	
**Syringe pump**	**Set flow rate (μL s** ^**−1**^ **)**	**100**	
**Syringe pump**	**Aspirate (μL)**	**50**	**(Luminol 5.0×10** ^**−4**^ **mol L** ^**−1**^ **)**
**Multiposition valve**	**Set valve position**	**2**	
**Syringe pump**	**Set flow rate (μL s** ^**−1**^ **)**	**100**	
**Syringe pump**	**Aspirate (μL)**	**50**	**(Gold nanoparticles 1.0×10** ^**−5**^ **mol L** ^**−1**^ **)**
**Multiposition valve**	**Set valve position**	**5**	
**Syringe pump**	**Set flow rate (μL s** ^**−1**^ **)**	**100**	
**Syringe pump**	**Aspirate (μL)**	**30**	**Sample (NLX)**
**Syringe pump**	**Delay until done**		
**Multiposition valve**	**Set valve position**	**4**	
**Syringe pump**	**Set flow rate (μL s** ^**−1**^ **)**	**100**	
**Syringe pump**	**Aspirate (μL)**	**30**	**(Potassium ferricyanide1.0×10** ^**−2**^ **mol L** ^**−1**^ **)**
**Syringe pump**	**Delay until done**		
**Multiposition valve**	**Detector**	**7**	
**Syringe pump**	**Set flow rate (μL s** ^**−1**^ **)**	**100**	
**PMT**	**Start scan**		
**Syringe pump**	**Empty**		
**Syringe pump**	**Delay until done**		
**PMT**	**Stop scans**		
**Refresh plat**			
**Loop end**			

### Characterizations

The developed SIA-CL method was used for determination of NLX in its bulk powder and pharmaceutical dosage forms. The enhancing effect of AuNPs on luminol-ferricyanide system gave high sensitivity and accurate results. As presented in Table 2 the results for determination of NLX in pure bulk and its pharmaceutical dosage form were 99.1±0.7 and 98.9±0.8 respectively. The obtained results were statistically treated *t*-test and F-test [20] and compared with those obtained from high performance liquid chromatography reported method [3]. The results did not reveal any significant difference between them at 95% confidence level proving similar accuracy and precision. The use of the SIA-CL technique is less time consuming and cost effective for reagents and samples and the developed method gave wide linear concentration ranges and more simple and sensitive than other reported methods.

**Table 2:** **Performance data obtained from the determination of NLX using AuNPs-luminol - potassium ferricyanide system**

**Analytical characteristics**	**Value**
Linear range, mol L^−1^	1.0×10^−9^-1.0×10^−2^
Detection limit, mol L^−1^	1.6×10^−11^
Quantification limit, mol L^−1^	1.0×10^−9^
Intercept on the ordinate	317858
Slope	25214.6
% RSD (n=9)	0.9%
Correlation coefficient, r	0.9993

### Effect of foreign substances

To study the effect of foreign substances on the selectivity of the proposed CL method for detection of NLX in its pharmaceutical dosage forms and some exicpients was investigated. Also, the influence of foreign substances such as sugars, amino acids and some common cations, the effect of related pharmacological active drugs were tested. 1.0×10^−3^ mol L^−1^ of NLX was investigated by adding an amount of foreign substances ≈ 1.0×10^−1^ mol L^−1^. The mean peak heights were compared with those obtained with 1.0×10^−3^ mol L^−1^ pure standard NLX. The tolerable level was defined as the amount of foreign species that produces an error not exceeding 5% in determining the investigated substance. The obtained results as indicated in Table 3 gave good selectivity for determination of the investigated NLX. It can be seen that there is no influence on the determination of NLX in pharmaceutical dosage forms. While, for serum samples main possible interference from ascorbic acid, uric acid, urea, heavy metal ions such as Fe^3+^, Fe^2+^, Cd^2+^, Co^2+^, Cu^2+^, Al^3+^ and Ni^2+^ was observed. These substances have a very important role in the vital activities in the body and may present in the human serum, so we should study the influence of such substances on the chemiluminescence analysis. We found that the presence of such substances during the analysis gave some interferences in the peaks hight. The latter ions can be eliminated by the addition of EDTA. After 1000 fold dilution the interference from such substances could be greatly minimized to negligible levels. Also, the recorded results showed interference due to the presence of some related pharmacological active drugs such as nalbuphine hydrochloride and naltrexone hydrochloride in the determination of NLX.

**Table 3:** **Tolerable concentration level of interferents with respect to NLX**

**Interferents**	**Tolerable level mol L** ^**−1**^
Na^+^, K^+^, Mg^2+^, Cl^−^, NO_3_^−^, NH_4_^+^, EDTA and SO_4_^2−^	4.8×10^−5^
Glucose, sucrose, lactose, talc, starch, magnesium stearate, citric acid	9.2×10^−6^
Uric acid, ascorbic acid and urea	5.6×10^−2^
Adrenaline, dopamine, cysteine, histamine, tyrosine, glucosamine	8.4×10^−5^
Al^3+^, Cd^2+^, Co^2+^, Fe^2+^, Fe^3+^, Mn^2+^, Ni^2+^, and Cu^2+^	2.6×10^−1^
Nalbuphine hydrochloride and naltroxone hydrochloride	1.5×10^−1^

### Method validation

Method validation is the process used to confirm that the analytical procedure employed for a specific test is suitable for its intended use. In the present study method validation was carried out with respect to linearity, lower limit of detection, quantification limit, accuracy, precision, and robustness according to ICH guidelines [21].

#### Linearity

The proposed SIA-CL method using AuNPs-luminol-ferricyanide system was successfully employed for determination of NLX. The linearity of the proposed method was recorded by plotting signals as a function of the tested drug concentrations. Nine standard solutions of NLX were subjected to SIA-CL detection. The regression line was calculated using the least square statistical method and was found to be (r = 0.999). The results obtained clarified that the proposed SIA-CL method exhibits linear concentration ranges at 1.0×10^−9^-1.0×10^−2^ mol L^−1^.

#### Lower limit of detection LOD

The lower limit of detection of the proposed SIA-CL method for determination of NL was evaluated using signal-to-noise ratio. It was detected by The lower limit of detection S/N=3 as the concentration of NLX has a CL signal equals three times that of a blank signal. The recorded signal showed the lower limit of detection 3.3×10^−10^ mol L^−1^.

#### Quantification limit

The quantification limit of NLX was determined by the proposed AuNPs- luminol- ferricyanide SIA-CL method using signal-to-noise ratio equal to 10. The evaluation of quantification limits (N= 10 σ/S) was found to be 1.0 ×10^−9^ mol L^−1^.

#### Accuracy

The accuracy of the developed AuNPs-luminol-ferricyanide SIA-CL method for determination of NLX was investigated using standard addition method. The accuracy was calculated in terms of mean percentage recovery. The tested NLX standard solutions were subjected to CL analysis and the calculated % recovery was found to be 99.25±0.84.

#### Precision

The method precision of the proposed AuNPs- luminol-ferricyanide SIA-CL method for determination of NLX was evaluated using intra-day and inter-day terms. Nine replicates were carried out in this study and the obtained results were calculated as % RSD values. The precision of the proposed SIA-CL method was found to be 0.7% and 0.6% of intra- and inter-day, respectively as shown in Table 4. The above % RSD value is less than 2% indicating good precision.

**Table 4:** **The recovery studies of the proposed SIA-CL method for the determination of NLX**

**Taken (−log conc. mol L** ^**−1**^ **)**	**Intra-day**		**Inter-day**	
	**Found (mol L** ^**−1**^ **)**	*Recovery %	**Found (mol L** ^**−1**^ **)**	**Recovery %**
9.0	8.99	99.9	8.97	99.7
8.3	8.27	99.6	8.24	99.3
8.0	7.97	99.6	7.92	99.0
7.0	6.95	99.3	6.92	98.9
6.0	5.94	99.0	5.91	98.5
5.0	4.92	98.4	4.96	99.2
4.0	3.92	98.0	3.97	99.3
3.0	2.94	98.0	2.99	99.7
2.0	1.99	99.5	1.96	98.0
Mean % ±SD		99.0±0.7		99.1±0.6
% RSD		0.7%		0.6%

*Values are mean of three determinations for intra and inter-day determination.

#### Robustness

The robustness of the SIA-CL method for determination of NLX was carried out by introducing a small change in method parameters. Flow rate, aspirate rate, reagents and sample volumes were changed using 100±10 μL s^−1^, 50±5 μL and 30±5 μL, respectively. The calculated % recovery of the proposed method was 99.12±0.5%. The obtained results were closely in agreement with those obtained from standard drug solutions.

### Analytical applications

The proposed SIA-CL method was employed for determination of NLX. It was evident from obtaining results that it gave satisfactory results for the determination of NLX in pure forms and its dosage forms Table 5. The obtained results were statistically treated *t*-test and F-test [20] and compared with those obtained from high performance liquid chromatography reported method [3]. The results did not reveal any significant difference between them at 95% confidence level proving similar accuracy and precision. On the other hand, the proposed method was employed to determine the investigated drug in biological fluids such as human urine and serum as shown in Table 6. The obtained results indicated wide linear concentration ranges and more simple and sensitive than other reported methods. Also, it is less time consuming and cost effective for reagents and samples.

**Table 5:** **Determination of NLX using AuNPs-luminol-potassium ferricyanide SIA-injection CL detection in pure samples and dosage forms in comparison with reported method** [3]

**Pure samples**			**Narcan® ampoules**			**Reported method [** 3 **]**		
**Taken-log conc.**	**Found-log conc.**	**Recovery %**	**Taken-log conc.**	**Found-log conc.**	**Recovery %**	**Taken-log conc.**	**Found-log conc.**	**Recovery %**
9.0	8.99	99.9	9.0	9.00	100.0	5.0	4.96	99.2
8.0	7.98	99.8	8.0	7.88	98.5	4.7	4.66	99.1
7.0	6.99	99.9	7.0	6.99	99.9	4.5	4.45	98.9
6.0	5.96	99.3	6.0	5.95	99.2	3.9	3.89	99.7
5.0	4.99	99.8	5.0	4.99	99.8	3.6	3.56	98.9
4.0	3.95	98.8	4.0	3.99	99.8	3.3	3.31	100.3
3.0	2.98	99.3	3.0	2.98	99.3			
2.0	2.00	100.0						
Mean %±SD		99.6±0.4	99.5±0.5			99.4±0.6		
n		8	7			6		
Variance		0.16	0.25			0.36		
% SE*		0.14	0.09			0.24		
% RSD		0.40	0.50			0.60		
*t*-test		0.72(2.179)**	0.39(2.201)**					
F-test		2.25(4.15)**	1.44(4.21)**					

*% SE= SD/√n; **Figures in parentheses are the tabulated values of t-and F-testes at 95% confidence limit [20].

**Table 6:** **Determination of NLX using AuNPs-luminol-potassium ferricyanide SIA-injection CL detection in serum and urine**

**Serum samples**			**Urine samples**		
**Taken-log conc.**	**Found-log conc.**	**Recovery %**	**Taken log conc.**	**Found-log conc.**	**Recovery %**
9.0	8.94	99.3	9.0	9.00	100.0
8.0	7.99	99.9	8.0	7.99	99.8
7.0	6.96	99.4	7.0	6.95	99.3
6.0	5.98	99.7	6.0	5.87	97.8
5.0	4.96	99.2	5.0	4.96	99.2
4.0	3.93	98.3	4.0	3.94	98.5
3.0	2.97	99.0	3.0	2.99	99.7
Mean %±SD		99.3±0.5	99.2±0.8		
n		7	7		
Variance		0.25	0.64		
% SE		0.19	0.30		
% RSD		0.50	0.80		

## Experimental

### Materials and reagents

All reagents were of analytical grade and were used without further purification. Distilled water was used throughout the experiments. Pure grade of naloxone hydrochloride 99.8% and Narcan® ampoules, each ampoule (1 mL) claimed to contain 400 μg mL^−1^ naloxone hydrochloride were kindly supplied by Bristol Myer Squibb Co., Egypt. 5.0×10^−4^ mol L^−1^ luminol (Sigma Chemical Co.) stock solution was prepared in 100 mL of 1.0×10^−2^ mol L^−1^ sodium hydroxide (WinLab). Potassium ferricyanide (WinLab) was used to prepare 1.0×10^−2^ mol L^−1^ solution by dissolving 0.33 g in 100 mL distilled water. Gold nanoparticles 1.0×10^−1^ mol L^−1^ were purchased from (Sigma-Aldrich -Germany). Urine samples were obtained from healthy volunteers and serum samples (Multi-Serum Normal, Randox Laboratories, UK) were obtained from commercial sources.

### Apparatus

The fluorimetric/chemiluminescence measurements were carried out using an SIA system (FIAlab-3500 instrument, USA). It comprises of a CAVRO XL 3000 syringe pump volume 2.5 mL (Cavro Scientific Instrument Int., USA) and Vici Valco Cheminer RT® 125–0718 eight-port manifolds. The detector used is UV-lamp equipped with lab-made CL module and the photomultiplier tube voltage was 320 V. The system was conneted with autosampler model ALM 3200. Moreover, SIA- system involving a holding coil (length 70 cm, i.d. 0.8 mm, PTFE tubing volume 1.2 mL). The recorded signals were PC controlled and (FIAlab for windows version 5.9.321) software was used for data acquisition.

### Preparation of samples

#### Standard drug solution

A stock standard NLX solution 1.0×10^−1^ mol L^−1^ was prepared by dissolving 1.82 g of pure drug in 50 mL distilled water. Serial solutions were prepared daily by appropriate dilution. The employed working solutions were in the range of 1.0×10^−10^-1.0×10^−1^ mol L^−1^.

#### Preparation of injection solution

The Contents of 10 Narcan® ampoules each containing 400 μg mL^−1^ aqueous NLX were mixed. 1 mL of the drug solution was transferred into a 10-mL volumetric flask and completed to volume with distilled water to obtain a solution labeled to contain 1.0×10^−3^ mol L^−1^ NLX. The working solutions were prepared by serial dilutions in the range of 1.0×10^−9^-1.0×10^−3^ mol L^−1^. The proposed SIA-CL method was employed to determine the investigated drug in each concentration. The mean % recoveries were calculated using a calibration graph.

#### Preparation of serum and urine solutions

The developed enhanced AuNPs-SIA-CL technique was employed for determination of NLX in human serum and urine. Spiking technique was used for the preparation of human serum and urine samples. 1.0 mL of serum was spiked with a NLX standard drug solution to contain 1.0×10^−1^ mol L^−1^ and deprotinated by adding 1.0 mL acetonitrile. Then 0.1 mL of NaOH (0.1 mol L^−1^), 1.0 mL of ZnSO_4_.7 H_2_O (5.0% w/v) were added, where most of the interfering species (mainly proteins) were removed by precipitation [22]. The prepared solution was centrifuged at 2500 rpm for 5 min. The treated sample was diluted with distilled water to obtain a concentration of NLX in the range of 1.0×10^−9^-1.0×10^−3^ mol L^−1^. No further pre-treatment was required for urine samples. AuNPs-SIA-CL detection was employed and the peak heights of CL signals were recorded and the % recovery was calculated by comparing the obtained results in serum and urine with the same concentration levels of the drug in the water.

#### Preparation of gold nanoparticles AuNPs solution

A standard solution of 1.0×10^−1^ mol L^−1^ AuNPs was purchased from Sigma-Aldrich Co. Working solutions were prepared by serial dilution using distilled water in the range of 1.0×10^−5^-1.0×10^−1^ mol L^−1^ to select the suitable concentration that gives the maximum SIA-CL signal.

### Procedure

**Figure 7:**
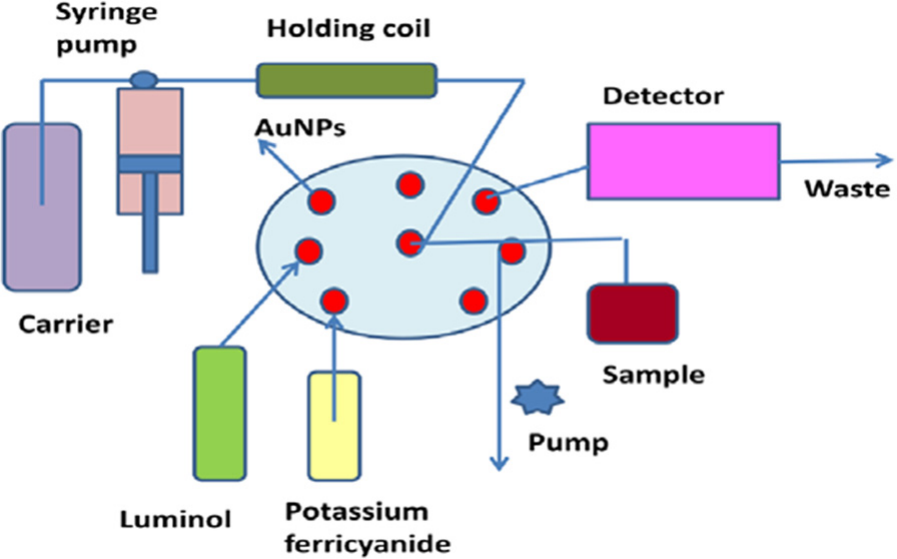
**Schematic diagram of SIA injection system for chemiluminescence determination of naloxone hydrochloride; carrier stream (water); reagent 1 (luminol 5.0×** **10** ^**−4**^ **mol L** ^**−1**^ **); reagent 2 (potassium ferricyanide 1.0×** **10** ^**−2**^ **mol L** ^**−1**^ **); reagent 3 (Au NPs 1.0×** **10** ^**−5**^ **mol L** ^**−1**^ **, sample (naloxone hydrochloride).**

All chemiluminescence measurements were introduced under PC control to ensure precision, control the valves and pump movements. To fill all lines of the system carrier solution was introduced at first to remove the air bubbles, then the test solution and reagents were aspirated as a mixture of of 50 μL of 5.0×10^−4^ moL L^−1^ luminol, 50 μL of 1.0×10^−5^ mol L^−1^ AuNPs, 30 μL sample solution and 30 μL of 1.0×10^−2^ mol L^−1^ potassium ferricyanide through the relevant ports of the multiposition valve at flow rate 100 μL s^−1^ as shown in Figure 7. The resulting emission of the reaction was monitored using flowthrough cell of PMT. The recorded signals were automatically calculated by (FIAlab® supported software, Version 5.9.321). All measurements were carried out at ambient temperature 25±1°C.

### Calibration

The calibration curve was plotted under the optimum conditions for determination of NLX. The graph related the CL intensity vs. the concentration of testing drug solutions was plotted at 9 experimental points. The mean peak heights were obtained after triplicate sample aspiration. Conventional linear regression was utilized for fitting the curve.

## Conclusion

A new sensitive and selective SIA-CL method for determination of NLX was developed. The developed method was employed to determine the investigated drug using the enhancement effect of AuNPs on luminol-potassium ferricyanide system. The enhanced effect of AuNPs in the presence of NLX was proportional to its concentration and the linear concentration range was found to be 1.0×10^−9^-1.0×10^−2^ mol L^−1^. The results obtained were treated statically and revealed good agreements with obtained from the reported method. The proposed method was introduced to determine NLX in bulk powder, its dosage forms and biological fluids.
